# Preoperative heart rate and myocardial injury after non-cardiac surgery: results of a predefined secondary analysis of the VISION study

**DOI:** 10.1093/bja/aew182

**Published:** 2016-07-20

**Authors:** T. E. F. Abbott, G. L. Ackland, R. A. Archbold, A. Wragg, E. Kam, T. Ahmad, A. W. Khan, E. Niebrzegowska, R. N. Rodseth, P. J. Devereaux, R. M. Pearse

**Affiliations:** 1 William Harvey Research Institute, Queen Mary University of London, London, UK; 2 Barts Health NHS Trust, London, UK; 3 Shaukat Khanum Memorial Cancer Hospital, Lahore, Pakistan; 4 Nelson R. Mandela School of Medicine, University of KwaZulu-Natal, Durban, South Africa; 5 Population Health Research Institute, McMaster University, Hamilton, Ontario, Canada

**Keywords:** heart rate, observational study, surgery

## Abstract

**Background:**

Increased baseline heart rate is associated with cardiovascular risk and all-cause mortality in the general population. We hypothesized that elevated preoperative heart rate increases the risk of myocardial injury after non-cardiac surgery (MINS).

**Methods:**

We performed a secondary analysis of a prospective international cohort study of patients aged ≥45 yr undergoing non-cardiac surgery. Preoperative heart rate was defined as the last measurement before induction of anaesthesia. The sample was divided into deciles by heart rate. Multivariable logistic regression models were used to determine relationships between preoperative heart rate and MINS (determined by serum troponin concentration), myocardial infarction (MI), and death within 30 days of surgery. Separate models were used to test the relationship between these outcomes and predefined binary heart rate thresholds.

**Results:**

Patients with missing outcomes or heart rate data were excluded from respective analyses. Of 15 087 patients, 1197 (7.9%) sustained MINS, 454 of 16 007 patients (2.8%) sustained MI, and 315 of 16 037 patients (2.0%) died. The highest heart rate decile (>96 beats min^−1^) was independently associated with MINS {odds ratio (OR) 1.48 [1.23–1.77]; *P*<0.01}, MI (OR 1.71 [1.34–2.18]; *P*<0.01), and mortality (OR 3.16 [2.45–4.07]; *P*<0.01). The lowest decile (<60 beats min^−1^) was independently associated with reduced mortality (OR 0.50 [0.29–0.88]; *P*=0.02), but not MINS or MI. The predefined binary thresholds were also associated with MINS, but more weakly than the highest heart rate decile.

**Conclusions:**

Preoperative heart rate >96 beats min^−1^ is associated with MINS, MI, and mortality after non-cardiac surgery. This association persists after accounting for potential confounding factors.

**Clinical trial registration:**

NCT00512109.

**Editor's key points**

- Increased baseline heart rate is associated with cardiovascular morbidity and mortality in the general population, but its impact on perioperative outcomes is unclear.
- Secondary analysis of an international multicentre study of patients undergoing non-cardiac surgery was performed.
- In this large prospective cohort study, heat rate >96 beats min^−1^ was associated with increased myocardial injury, myocardial infarction, and mortality.

More than 310 million surgical procedures are carried out worldwide each year, with mortality rates of up to 4%.^1,2^ Cardiovascular complications are a prominent cause of postoperative morbidity and mortality.^3^ Recent data suggest that one in 10 surgical patients experience postoperative myocardial injury attributable to ischaemia, characterized by a transient increase in the serum concentration of cardiac troponin, which is strongly associated with 30 day death.^4,5^ In contrast to other acute cardiac events, perioperative myocardial injury is predominantly asymptomatic, and in the absence of routine surveillance of troponin concentrations, four out of five instances are not identified.^4,6^ Conventional teaching suggests that tachycardia is an important causal factor for myocardial injury, as a result of myocardial oxygen supply–demand imbalance.^7,8^ Epidemiological data from the general population consistently demonstrate that resting heart rate is associated with cardiovascular risk and mortality.^9–12^ These relationships appear independent of underlying pathology or cardiorespiratory fitness.^9–13^ Consequently, there has been widespread interest in therapies to control heart rate, both in general medical and perioperative patients.^13,14^

A number of trials have demonstrated that perioperative treatment with β-blockers, to lower heart rate, reduces the risk of perioperative myocardial infarction (MI).^14,15^ However, the results of the largest clinical trial demonstrated that β-block increased the risk of mortality.^15^ This has led to the suggestion that the optimal dose of β-blocker varies, and that preoperative heart rate might be used to determine the appropriate dosage in individual patients.^16^ However, this remains controversial, and the association between preoperative heart rate and postoperative cardiac complications has been explored in only a small number of studies.^17–20^ Except for the POISE Trial, none of these involved the routine measurement of cardiac biomarkers to identify myocardial injury, and statistical analyses used arbitrary predefined heart rate thresholds.^15,18–20^ Thus, it remains unclear whether or not there is an increased relationship between preoperative heart rate and myocardial injury, or if there is a heart rate threshold at which the risk of postoperative myocardial injury increases.

The aim of this analysis was to assess, in the perioperative setting, the relationships between heart rate and cardiovascular outcomes previously described in general medical patients. We hypothesized that elevated preoperative heart rate is associated with increased risk of myocardial injury, MI, and mortality within 30 days of surgery.

## Methods

### Study design

This was a predefined secondary analysis of data prospectively collected in the Vascular Events in Non-cardiac Surgery Patients Cohort Evaluation (VISION) study, an international prospective observational cohort study of clinical outcomes after non-cardiac surgery. The full methods have been published previously.^4,5^ Research ethics committees or boards approved the study at each site before patient recruitment commenced. The study was registered with ClinicalTrials.gov (NCT00512109).

### Patient population

Patients aged 45 yr or older undergoing non-cardiac surgery under regional or general anaesthesia with an expected overnight hospital stay were eligible for inclusion. Patients gave written informed consent before surgery or, where this was not possible (e.g. emergency surgery), consent was obtained within 24 h after surgery. Eight hospitals used a deferred consent process for patients who could not provide consent and for whom no next of kin was available.^4,5^ Patients were excluded if they refused consent or if they had been previously enrolled in the study.

### Conduct of the study

A detailed data set was collected before and 30 days after surgery (definitions of the variables are reported in the Supplementary file). Researchers collected data from patients and their medical notes. Preoperative heart rate was measured as part of routine care at each site and was defined as the last heart rate measurement recorded before induction of anaesthesia. Blood was sampled between 6 and 12 h after surgery and on days 1, 2, and 3 after surgery. Serum troponin T (TnT) concentration was measured using a Roche Diagnostics (Basel, Switzerland) fourth generation Elecsys™ assay. If TnT was ≥0.04 ng ml^−1^ (the widely accepted laboratory reference value at the start of the study), an ECG was performed. In the absence of dynamic ECG findings or clinical features of myocardial ischaemia, clinicians were encouraged to obtain an echocardiogram.

### Outcome measures

The primary outcome measure was myocardial injury after non-cardiac surgery (MINS), defined as TnT ≥0.03 ng ml^−1^, adjudicated as attributable to an ischaemic pathology within 30 days after surgery. Non-ischaemic causes of TnT elevation (e.g. sepsis, pulmonary embolism) were excluded. This definition and TnT threshold was previously defined using VISION data and is the definition of MINS recommended by the European Society of Anaesthesia and European Society of Intensive Care Medicine joint taskforce on clinical outcomes in perioperative medicine.^4,21^ Secondary outcome measures were MI and death within 30 days of surgery. Myocardial infarction was defined according to the third universal definition (troponin elevation in the presence of at least one of the following: ischaemic symptoms; new or presumed new Q waves, ST segment or T wave changes, or left bundle branch block on the electrocardiogram; or new or presumed new regional wall motion abnormality on echocardiography).^21^ Patients with a troponin elevation <0.04 ng ml^−1^ were not investigated for evidence of ischaemia.

### Statistical analysis

We used SPSS version 22 (IBM, New York, NY, USA) for the main statistical analysis. We ranked the sample according to integer values of preoperative heart rate and divided the sample into deciles, using cut-points closest to each 10th percentile, with approximately equal numbers of patients in each group. As a result of the distribution of patients, some groups contained more or fewer patients than average. The groups were compared for differences in baseline characteristics. We constructed multivariable logistic regression models for heart rate against each outcome measure, considering each decile as a categorical variable. We used deviation contrasts to compare each heart rate category with the unweighted average effect for the whole cohort because we did not want to isolate any particular heart rate decile as a reference category.^22,23^ We corrected each multivariable model for covariates that were previously associated with MINS, cardiac events (including MI), or mortality in other perioperative epidemiological research, as follows: age (45–64, 65–75, or >75 yr), current atrial fibrillation, diabetes mellitus, hypertension, heart failure, coronary artery disease, peripheral vascular disease, previous stroke or transient ischaemic attack, estimated glomerular filtration rate (<30, 30–44, 45–60, or >60 ml min^−1^), chronic obstructive pulmonary disease, neurosurgery, major surgery, and urgent or emergency surgery; these were considered as categorical variables in the multivariable models.^4,5,24,25^ Full definitions are listed in the Supplementary file. Missing data were handled by list-wise deletion. The results of multivariable logistic regression analyses are presented as odds ratios (ORs) with 95% confidence intervals. Normally distributed data are expressed as the mean (sd), and non-normally distributed data are expressed as the median (interquartile range). Binary data are expressed as percentages.

### Secondary analyses

Previous studies have investigated heart rate as a risk factor for cardiac complications using predefined heart rate thresholds. To allow comparisons between our findings and previous research, we repeated our analysis using two heart rate thresholds that were associated with MI or mortality in the general medical literature (>70 beats min^−1^) and the perioperative literature (>104 beats min^−1^).^8,26^ We dichotomized the sample according to each heart rate threshold and constructed multivariable logistic regression models for each outcome measure, corrected for the previous covariates. Heart rate above the threshold was considered as a categorical variable.

### Sensitivity analyses

We repeated the multivariable logistic regression analyses using a single heart rate decile as the reference category, rather than the whole cohort. To determine the influence of preoperative atrial fibrillation, we excluded all patients with a history of atrial fibrillation and repeated the logistic regression analyses. Secondly, to determine the influence of emergency surgery, we excluded all emergency surgery patients and repeated the logistic regression analyses. It is plausible that an observed relationship between heart rate and one or more of the outcome measures could be confounded by the use of medications that influence heart rate. The most relevant agents in clinical practice are β-adrenoceptor antagonists (β-blockers) and the negatively chronotropic calcium channel blockers, diltiazem and verapamil. To determine the influence of these agents, we conducted a *post hoc* analysis by excluding patients who received a β-blocker, a rate-limiting calcium channel blocker, or both within 24 h before surgery and repeating the primary statistical analysis. To investigate the possibility of a non-linear relationship between heart rate and myocardial injury in more detail, we conducted a *post hoc* analysis using multivariable fractional polynomial regression. This technique fits a set of power functions (3, 2, 1, 0.5, 0, −0.5, −1, and −2, where 0 represents the natural logarithm) to continuous variables within the model.^27,28^ We used STATA version 14 (StataCorp LP, College Station, TX, USA) to fit the most efficient polynomial model to our data and then repeated a logistic regression analysis using the polynomial functions of the independent variables.

## Results

**Fig 1:**
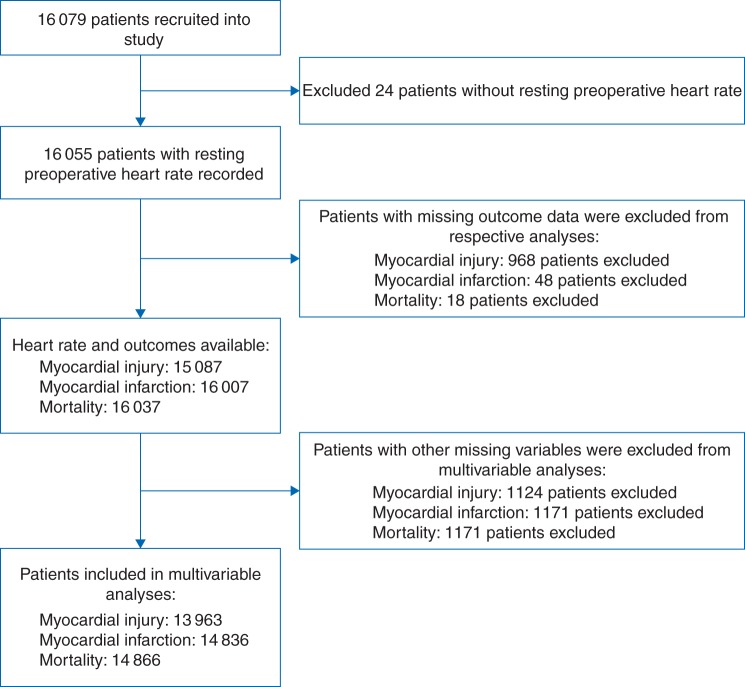
Patient flow diagram showing the number of patients included and excluded from each analysis.

A total of 16 079 patients were recruited to the VISION study from 12 hospitals in eight countries between August 6, 2007 and January 11, 2011.^4^ We excluded patients with missing data describing preoperative heart rate or patient outcomes. Of 15 087 patients, 1197 (7.9%) sustained MINS, 454 of 16 007 patients (2.8%) sustained MI, and 315 of 16 037 patients (2.0%) died, within 30 days of surgery. Patients who were missing predefined covariates were excluded from multivariable analyses (Fig. 1). Baseline characteristics are presented in Table 1. There was a clear increase in the incidences of the outcome measures for heart rates >96 beats min^−1^. The highest heart rate decile was associated with increased incidences of preoperative atrial fibrillation (*P*<0.01), diabetes mellitus (*P*<0.01), peripheral vascular disease (*P*<0.01), previous stroke or transient ischaemic attack (*P*<0.01), chronic obstructive pulmonary disease (*P*=0.02), estimated glomerular filtration rate <30 ml min^−1^ (*P*<0.01), and estimated glomerular filtration rate 30–44 ml min^−1^ (*P*<0.01).

**Table 1:** Baseline patient characteristics. Descriptive data are stratified by preoperative heart rate decile, presented as frequencies with percentages (%) or mean (sd). Data are rounded to the nearest whole number

Characteristic	Preoperative resting heart rate deciles (beats min^−1^)										
	Whole cohort	<60	60–64	65–68	69–71	72–74	75–79	80–82	83–87	88–96	>96
Number of patients (*n*)	16 055	1515	1676	1579	1464	1318	2019	1649	1352	1989	1494
Age [yr; mean (sd)]	65 (53–77)	66 (5–77)	66 (55–77)	66 (54–78)	64 (52–76)	65 (53–77)	65 (53–77)	65 (53–77)	65 (53–77)	65 (52–78)	65 (52–78)
Sex											
Male [*n* (%)]	7739 (48)	915 (60)	888 (53)	795 (50)	704 (48)	634 (48)	914 (45)	784 (48)	578 (43)	871 (44)	667 (45)
Female [*n* (%)]	8316 (52)	600 (40)	788 (47)	784 (50)	760 (52)	684 (52)	1105 (55)	865 (52)	774 (57)	1118 (56)	827 (55)
Preoperative heart rate [beats min^−1^; mean (sd)]	77 (15)	54 (5)	62 (2)	67 (1)	70 (1)	73 (1)	77 (1)	81 (1)	85 (1)	91 (3)	107 (11)
Preoperative systolic arterial pressure [mm Hg; mean (sd)]	140 (24)	137 (24)	138 (24)	137 (24)	136 (22)	140 (23)	139 (23)	141 (24)	140 (23)	144 (23)	145 (25)
Preoperative arterial pulse pressure [mm Hg; mean (sd)]	61 (19)	62 (20)	62 (20)	61 (20)	59 (19)	61 (19)	60 (19)	61 (19)	60 (19)	62 (19)	61 (20)
Co-morbid disorder [*n* (%)]											
Atrial fibrillation	545 (3)	46 (3)	44 (3)	39 (2)	46 (3)	32 (2)	56 (3)	67 (4)	39 (3)	79 (4)	97 (6)
Diabetes mellitus	3153 (20)	258 (17)	294 (18)	281 (18)	278 (19)	243 (18)	411 (20)	341 (21)	264 (20)	422 (21)	355 (24)
Hypertension	8171 (51)	808 (53)	887 (53)	791 (50)	693 (47)	636 (48)	1031 (51)	848 (51)	686 (51)	1006 (51)	771 (52)
Coronary artery disease	1947 (12)	284 (19)	256 (15)	227 (14)	182 (12)	133 (10)	215 (11)	172 (10)	145 (11)	175 (9)	153 (10)
Peripheral vascular disease	858 (5)	71 (5)	106 (6)	87 (6)	77 (5)	59 (5)	116 (6)	78 (5)	56 (4)	106 (5)	102 (7)
Previous stroke or transient ischaemic attack	1167 (7)	123 (8)	121 (7)	107 (7)	101 (7)	89 (7)	134 (7)	108 (7)	77 (6)	164 (8)	140 (9)
Chronic obstructive pulmonary disease	1337 (8)	116 (7)	128 (8)	118 (7)	120 (8)	108 (8)	156 (8)	147 (9)	112 (8)	180 (9)	149 (10)
Active cancer	2031 (13)	176 (12)	213 (13)	193 (12)	188 (13)	195 (15)	234 (12)	231 (14)	180 (13)	218 (11)	199 (13)
Preoperative estimated glomerular filtration rate [ml min^−^^1^; *n* (%)]											
<30	564 (4)	50 (4)	48 (3)	38 (3)	41 (3)	45 (4)	65 (4)	59 (4)	38 (3)	87 (5)	93 (7)
30–44	831 (5)	81 (6)	81 (5)	76 (5)	71 (5)	55 (4)	111 (6)	72 (5)	64 (5)	114 (6)	104 (7)
45–60	1579 (10)	166 (12)	165 (11)	176 (12)	145 (11)	106 (9)	175 (9)	166 (11)	135 (11)	198 (11)	145 (10)
>60	11 938 (74)	1105 (79)	1267 (81)	1157 (80)	1108 (81)	1020 (83)	1505 (8)	1216 (80)	1014 (81)	1467 (79)	1060 (76)
Surgical procedure category [*n* (%)]											
Elective	13 745 (86)	1377 (91)	1530 (91)	1413 (89)	1304 (89)	1149 (87)	1776 (88)	1409 (85)	1118 (83)	1585 (80)	1084 (73)
Urgent	483 (3)	28 (2)	35 (2)	28 (2)	33 (2)	30 (2)	45 (2)	44 (3)	51 (4)	80 (4)	109 (7)
Emergency	1826 (11)	110 (7)	111 (7)	138 (9)	126 (9)	139 (11)	198 (10)	196 (12)	183 (14)	324 (16)	301 (20)
Major surgery [*n* (%)]	9576 (60)	868 (57)	946 (56)	905 (57)	817 (56)	798 (61)	1202 (60)	980 (59)	846 (63)	1253 (63)	969 (65)
Outcome measures [*n* (%)]											
Myocardial injury	1197 (8)	103 (7)	105 (7)	100 (7)	100 (7)	70 (6)	131 (7)	117 (8)	119 (9)	169 (9)	177 (13)
Myocardial infarction	454 (3)	35 (2)	34 (2)	39 (3)	43 (3)	25 (2)	53 (3)	45 (3)	39 (3)	55 (3)	84 (6)
Mortality	315 (2)	13 (1)	19 (1)	16 (1)	22 (2)	14 (1)	34 (2)	30 (2)	22 (2)	52 (3)	91 (7)

**Fig 2:**
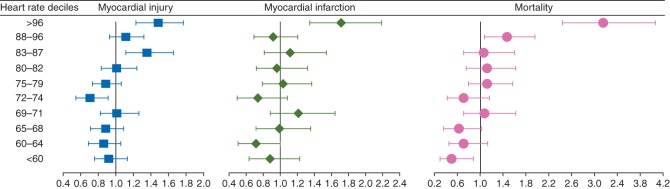
Forest plot showing the odds ratios for myocardial injury, myocardial infarction, and mortality by each heart rate (HR) decile (in beats per minute), with 95% confidence intervals. The odds ratios for myocardial injury are as follows: HR <60, 0.92 (0.76–1.14); HR 60–64, 0.86 (0.69–1.06); HR 65–68, 0.88 (0.71–1.09); HR 69–71, 1.01 (0.82–1.26); HR 72–74, 0.71 (0.55–0.91); HR 75–79, 0.88 (0.73–1.07); HR 80–82, 1.01 (0.83–1.24); HR 83–87, 1.36 (1.11–1.66); HR 88–96, 1.11 (0.93–1.32); and HR >96, 1.48 (1.23–177). The odds ratios for myocardial infarction are as follows: HR <60, 0.89 (0.64–1.23); HR 60–64, 0.72 (0.51–1.01); HR 65–68, 0.99 (0.72–1.36); HR 69–71, 1.21 (0.89–1.64); HR 72–74, 0.74 (0.50–1.09); HR 75–79, 1.04 (0.79–1.37); HR 80–82, 0.97 (0.72–1.32); HR 83–87, 1.12 (0.82–1.54); HR 88–96, 0.92 (0.70–1.21); and HR >96, 1.71 (1.34–2.18). The odds ratios for mortality are as follows: HR <60, 0.50 (0.29–0.88); HR 60–64, 0.71 (0.45–1.13); HR 65–68, 0.61 (0.37–1.02); HR 69–71, 1.07 (0.71–1.63); HR 72–74, 0.70 (0.42–1.17); HR 75–79, 1.12 (0.79–1.58); HR 80–82, 1.11 (0.76–1.62); HR 83–87, 1.06 (0.70–1.61); HR 88–96, 1.46 (1.08–1.97); and HR >96, 3.16 (2.45–4.07).

The results of multivariable logistic regression analyses for preoperative heart rate deciles as categorical variables against MINS, MI, and mortality within 30 days of surgery are reported in Table 2 and Fig. 2. Most of the associations observed on univariate analysis remained significant on multivariate analysis. In particular, heart rates in the highest decile (>96 beats min^−1^) were associated with MINS (OR 1.48 [1.23–1.77]; *P* <0.01), MI (OR 1.71 [1.34–2.18]; *P* <0.01), and mortality (OR 3.16 [2.45–4.07]; *P*<0.01). Heart rates in the ninth decile (88–96 beats min^−1^) were also associated with mortality (OR 1.46 [1.08–1.97]; *P*=0.01), but not with MINS or MI. Heart rates in the eighth decile (83–87 beats min^−1^) and fifth decile (73–74 beats min^−1^) were associated with MINS (OR 1.36 [1.11–1.66]; *P*<0.01 and OR 0.71 [0.55–0.91]; *P*=0.01, respectively), but not with MI or mortality. Heart rates in the lowest decile (<60 beats min^−1^) were associated with lower mortality rates than other deciles (OR 0.50 [0.29–0.88]; *P*=0.02), but this group was not associated with MINS or MI (Fig. 2). For comparison, we present the results of univariable logistic regression analysis of heart rate deciles against the outcome measures, showing unadjusted ORs in Table 3.

**Table 2:** Multivariable logistic regression models for preoperative heart rate deciles. Dependent variables are myocardial injury, myocardial infarction, and mortality within 30 days of surgery. Preoperative heart rate was stratified by decile. Each decile was compared with the unweighted average heart rate for the whole sample

Covariates	Myocardial injury		Myocardial infarction		Mortality	
	Odds ratio	*P*-value	Odds ratio	*P*-value	Odds ratio	*P*-value
Age (yr)						
45–64 (reference)	–	–	–	–	–	–
65–75	1.08 (0.90–1.30)	0.43	1.16 (0.87–1.55)	0.30	1.64 (1.17–2.30)	<0.01
>75	2.08 (1.74–2.48)	<0.01	1.90 (1.45–2.49)	<0.01	2.41 (1.73–3.35)	<0.01
Male sex	1.40 (1.22–1.61)	<0.01	1.04 (0.85–1.28)	0.70	1.27 (0.99–1.63)	0.06
History of atrial fibrillation	1.53 (1.18–2.00)	<0.01	1.29 (0.90–1.85)	0.17	0.97 (0.60–1.56)	0.89
History of diabetes mellitus	1.39 (1.19–1.61)	<0.01	1.21 (0.97–1.52)	0.10	0.98 (0.73–1.32)	0.91
History of hypertension	1.31 (1.12–1.54)	<0.01	1.41 (1.10–1.80)	0.01	0.98 (0.75–1.29)	0.89
History of heart failure	1.59 (1.26–1.99)	<0.01	1.67 (1.24–2.25)	<0.01	1.38 (0.92–2.10)	0.12
History of coronary artery disease	1.48 (1.25–1.76)	<0.01	2.23 (91.77–2.81)	<0.01	0.96 (0.68–1.34)	0.80
History of peripheral vascular disease	2.17 (1.77–2.65)	<0.01	2.11 (1.60–2.78)	<0.01	1.75 (1.21–2.53)	<0.01
History of stroke or transient ischaemic attack	1.46 (1.20–1.78)	<0.01	1.14 (0.85–1.52)	0.37	1.53 (1.10–2.15)	0.01
Preoperative estimated glomerular filtration rate (ml min^−1^)						
<30	10.75 (8.69–13.29)	<0.01	3.98 (2.96–5.36)	<0.01	2.95 (2.01–4.31)	<0.01
30–44	2.51 (2.02–3.19)	<0.01	1.69 (1.22–2.34)	<0.01	1.58 (1.07–2.36)	0.02
45–60	1.68 (1.39–2.03)	<0.01	1.40 (1.04–1.87)	0.02	0.94 (0.63–1.40)	0.77
>60 (reference)	–	–	–	–	–	–
History of chronic obstructive pulmonary disease	1.18 (0.97–1.45)	0.10	1.17 (0.83–1.49)	0.46	1.93 (1.40–2.65)	<0.01
Neurosurgery	1.14 (0.87–1.51)	0.34	0.58 (0.35–0.98)	0.04	1.82 (1.71–2.82)	0.01
Urgent or emergency surgery	1.82 (1.54–2.15)	<0.01	2.14 (1.70–2.69)	<0.01	3.11 (2.41–4.02)	<0.01
Major surgery	1.66 (1.42–1.93)	<0.01	2.19 (1.71–2.80)	<0.01	1.51 (1.13–2.02)	0.01
Preoperative heart rate (beats min^−1^)						
<60	0.92 (0.75–1.14)	0.46	0.89 (0.64–1.23)	0.48	0.50 (0.29–0.88)	0.02
60–64	0.86 (0.69–1.06)	0.15	0.72 (0.51–1.01)	0.06	0.71 (0.45–1.13)	0.15
65–68	0.88 (0.71–1.09)	0.26	0.99 (0.72–1.36)	0.96	0.61 (0.37–1.02)	0.06
69–71	1.01 (0.82–1.26)	0.91	1.21 (0.89–1.64)	0.23	1.07 (0.71–1.63)	0.74
72–74	0.71 (0.55–0.91)	0.01	0.74 (0.50–1.09)	0.13	0.70 (0.42–1.17)	0.18
75–79	0.88 (0.73–1.07)	0.21	1.04 (0.79–1.37)	0.79	1.12 (0.79–1.58)	0.53
80–82	1.01 (0.83–1.24)	0.90	0.97 (0.72–1.32)	0.85	1.11 (0.76–1.62)	0.58
83–87	1.36 (1.11–1.66)	<0.01	1.12 (0.82–1.54)	0.47	1.06 (0.70–1.61)	0.78
88–96	1.11 (0.93–1.32)	0.23	0.92 (0.70–1.21)	0.92	1.46 (1.08–1.97)	0.01
>96	1.48 (1.23–1.77)	<0.01	1.71 (1.34–2.18)	<0.01	3.16 (2.45–4.07)	<0.01

**Table 3:** Univariable (unadjusted) logistic regression models for preoperative heart rate deciles. Dependent variables are myocardial injury, myocardial infarction, and mortality within 30 days of surgery. Preoperative heart rate was stratified by decile. Each decile was compared with the unweighted average heart rate for the whole sample

Heart rate deciles	Myocardial injury		Myocardial infarction		Mortality	
	Odds ratio	*P*-value	Odds ratio	*P*-value	Odds ratio	*P*-value
<60	0.92 (0.76–1.11)	0.40	0.85 (0.62–1.17)	0.31	0.52 (0.32–0.87)	0.01
60–64	0.85 (0.70–1.02)	0.08	0.74 (0.54–1.02)	0.07	0.69 (0.45–1.06)	0.09
65–68	0.86 (0.71–1.04)	0.11	0.91 (0.67–1.23)	0.54	0.62 (0.39–0.98)	0.04
69–71	0.94 (0.77–1.14)	0.51	1.09 (0.81–1.45)	0.57	0.92 (0.62–1.38)	0.70
72–74	0.71 (0.57–0.89)	<0.01	0.70 (0.48–1.01)	0.05	0.65 (0.40–1.06)	0.09
75–79	0.88 (0.75–1.05)	0.15	0.97 (0.75–1.26)	0.82	1.04 (0.74–1.44)	0.83
80–82	0.98 (0.82–1.17)	0.80	1.01 (0.76–1.34)	0.95	1.12 (0.79–1.59)	0.52
83–87	1.21 (1.01–1.45)	0.04	1.07 (0.79–1.45)	0.66	1.00 (0.67–1.49)	0.99
88–96	1.19 (1.02–1.40)	0.03	1.03 (0.79–1.33)	0.85	1.63 (1.23–2.15)	<0.01
>96	1.80 (1.54–2.10)	<0.01	2.14 (1.72–2.67)	<0.01	3.92 (3.11–4.94)	<0.01

### Secondary analyses

We repeated the multivariable analysis for two binary heart rate thresholds. Heart rates >104 beats min^−1^ were associated with MINS (OR 1.38 [1.21–1.57]; *P*<0.01), MI (OR 1.35 [1.14–1.61]; *P*<0.01), and mortality (OR 1.89 [1.60–2.24]; *P*<0.01), as shown in Table 4. Heart rates >70 beats min^−1^ were associated with MINS (OR 1.09 [1.01–1.17]; *P*=0.02) and mortality (OR 1.52 [1.30–1.77]; *P*<0.01), but not MI (Table 5).

**Table 4:** Preoperative heart rate threshold of 104 beats min^−1^. Multivariable logistic regression models for myocardial injury, myocardial infarction, and mortality, all within 30 days of non-cardiac surgery. Preoperative heart rate was stratified according to a threshold of 104 beats min^−1^

Covariates	Myocardial injury		Myocardial infarction		Mortality	
	Odds ratio	*P*-value	Odds ratio	*P*-value	Odds ratio	*P*-value
Age (yr)						
45–64 (reference)	–	–	–	–	–	–
65–75	1.07 (0.89–1.29)	0.47	1.15 (0.87–1.54)	0.32	1.60 (1.14–2.24)	<0.01
>75	2.05 (1.72–2.45)	<0.01	1.88 (1.44–2.47)	<0.01	2.37 (1.70–3.29)	<0.01
Male sex	1.38 (1.20–1.58)	<0.01	1.02 (0.83–1.26)	0.83	1.21 (0.95–1.55)	0.12
History of atrial fibrillation	1.53 (1.18–2.00)	<0.01	1.30 (0.91–1.86)	0.16	0.97 (0.60–1.57)	0.91
History of diabetes mellitus	1.40 (1.20–1.63)	<0.01	1.22 (0.98–1.53)	0.08	1.01 (0.75–1.35)	0.97
History of hypertension	1.32 (1.13–1.55)	<0.01	1.40 (1.09–1.78)	<0.01	0.99 (0.75–1.31)	0.95
History of heart failure	1.60 (1.27–2.00)	<0.01	1.69 (1.26–2.28)	<0.01	1.40 (0.92–2.11)	0.11
History of coronary artery disease	1.45 (1.23–1.72)	<0.01	2.19 (1.74–2.76)	<0.01	0.87 (0.63–1.22)	0.43
History of peripheral vascular disease	2.15 (1.75–2.63)	<0.01	2.11 (1.60–2.78)	<0.01	1.77 (1.23–2.55)	<0.01
History of stroke or transient ischaemic attack	1.45 (1.20–1.77)	<0.01	1.14 (0.85–1.51)	0.39	1.53 (1.10–2.14)	0.01
Preoperative estimated glomerular filtration rate (ml min^−1^)						
<30	10.69 (8.66–13.21)	<0.01	3.96 (2.94–5.32)	<0.01	2.97 (2.03–4.35)	<0.01
30–45	2.50 (2.01–3.10)	<0.01	1.70 (1.23–2.34)	<0.01	1.57 (1.06–2.34)	0.03
45–60	1.68 (1.39–2.03)	<0.01	1.40 (1.05–1.88)	0.02	0.94 (0.63–1.39)	0.74
>60 (reference)	–	–	–	–	–	–
History of chronic obstructive pulmonary disease	1.21 (0.99–1.47)	0.07	1.13 (0.85–1.51)	0.40	2.04 (1.49–2.80)	<0.01
Neurosurgery	1.14 (0.86–1.50)	0.36	0.58 (0.35–0.98)	0.04	1.75 (1.13–2.70)	0.01
Urgent or emergency surgery	1.88 (1.59–2.21)	<0.01	2.22 (1.77–2.79)	<0.01	3.50 (2.72–4.50)	<0.01
Major surgery	1.67 (1.43–1.95)	<0.01	2.19 (1.71–2.80)	<0.01	1.56 (1.17–2.08)	<0.01
Heart rate >104 beats min^−1^	1.38 (1.21–1.57)	<0.01	1.35 (1.14–1.61)	<0.01	1.89 (1.60–2.24)	<0.01

**Table 5:** Preoperative heart rate threshold of 70 beats min^−1^. Multivariable logistic regression models for myocardial injury, myocardial infarction, and mortality, all within 30 days of non-cardiac surgery. Preoperative heart rate was stratified according to a threshold of 70 beats min^−1^

Covariates	Myocardial injury		Myocardial infarction		Mortality	
	Odds ratio	*P*-value	Odds ratio	*P*-value	Odds ratio	*P*-value
Age (yr)						
45–64 (reference)	–	–	–	–	–	–
65–75	1.07 (0.89–1.28)	0.49	1.15 (0.86–1.53)	0.35	1.58 (1.13–2.20)	<0.01
>75	2.03 (1.70–2.42)	<0.01	1.85 (1.41–2.42)	<0.01	2.27 (1.63–3.15)	<0.01
Male sex	1.38 (1.20–1.59)	<0.01	1.03 (0.84–1.26)	0.81	1.26 (0.98–1.61)	0.07
History of atrial fibrillation	1.61 (1.24–2.09)	<0.01	1.37 (0.96–1.95)	0.09	1.08 (0.67–1.74)	0.74
History of diabetes mellitus	1.39 (1.20–1.62)	<0.01	1.22 (0.98–1.53)	0.08	0.99 (0.74–1.33)	0.96
History of hypertension	1.31 (1.12–1.53)	<0.01	1.38 (1.08–1.77)	0.01	0.97 (0.74–1.28)	0.84
History of heart failure	1.58 (1.26–1.99)	<0.01	1.67 (1.24–2.25)	<0.01	1.37 (0.91–2.07)	0.14
History of coronary artery disease	1.47 (1.24–1.74)	<0.01	2.20 (1.74–2.77)	<0.01	0.94 (0.67–1.31)	0.70
History of peripheral vascular disease	2.15 (1.76–2.63)	<0.01	2.11 (1.60–2.79)	<0.01	1.78 (1.24–2.57)	<0.01
History of stroke or transient ischaemic attack	1.47 (1.21–1.78)	<0.01	1.14 (0.85–1.52)	0.38	1.55 (1.11–2.16)	0.01
Preoperative estimated glomerular filtration rate (ml min^−1^)						
<30	10.83 (8.77–13.39)	<0.01	4.06 (3.02–5.46)	<0.01	3.17 (2.17–4.62)	<0.01
30–45	2.54 (2.05–3.15)	<0.01	1.75 (1.27–2.41)	<0.01	1.72 (1.16–2.54)	<0.01
45–60	1.69 (1.40–2.05)	<0.01	1.41 (1.05–1.89)	0.02	0.97 (0.65–1.44)	0.87
>60 (reference)	–	–	–	–	–	–
History of chronic obstructive pulmonary disease	1.20 (0.98–1.46)	0.08	1.13 (0.84–1.51)	0.42	1.95 (1.42–2.67)	<0.01
Neurosurgery	1.14 (0.86–1.50)	0.36	0.58 (0.34–0.98)	0.04	1.79 (1.16–2.76)	<0.01
Urgent or emergency surgery	1.91 (1.62–2.25)	<0.01	2.30 (1.83–2.89)	<0.01	3.53 (2.75–4.54)	<0.01
Major surgery	1.66 (1.43–1.94)	<0.01	2.19 (1.71–2.81)	<0.01	1.55 (1.16–2.07)	<0.01
Heart rate >70 beats min^−1^	1.09 (1.01–1.17)	0.02	1.06 (0.95–1.18)	0.29	1.52 (1.30–1.77)	<0.01

### Sensitivity analyses

Our principal findings remained similar when we repeated the regression analyses using a single decile (60–64 beats min^−1^) as the reference category (Supplementary data, Table S1), except that heart rates in the lowest decile were no longer associated with postoperative mortality (OR 0.71 [0.33–1.54]; *P*=0.39), heart rates 72–74 beats min^−1^ were no longer associated with myocardial injury (OR 0.83 [0.58–1.17]; *P*=0.28), and heart rates 69–71 beats min^−1^ were associated with MI (OR 1.68 [1.03–2.73]; *P*=0.04). To examine the potential confounding effect of preoperative tachyarrhythmia, we repeated the regression analyses excluding patients with current atrial fibrillation, the most common preoperative arrhythmia. When we excluded patients with current atrial fibrillation or emergency surgery, the results were very similar to the main results (Supplementary data, Tables S2–7).

**Fig 3:**
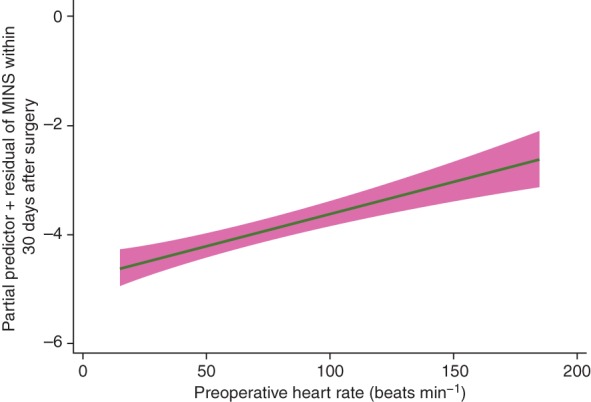
Plot of the fractional polynomial logistic regression model. The *x*-axis shows heart rate, and the *y*-axis is the probability of myocardial injury after non-cardiac surgery (MINS) within 30 days of surgery.

The association between the highest decile of heart rate and each of the three outcome measures was not affected by exclusion of patients who received a β-blocker, a rate-limiting calcium channel blocker, or both within 24 h before surgery (Supplementary data, Table S8). The ORs for MINS, MI, and 30 day mortality were 1.52 (1.24–1.85; *P*<0.01), 1.83 (1.38–24.1; *P*<0.01), and 2.90 (2.19–3.84; *P*<0.01), respectively. However, heart rates <60 beats min^−1^ were no longer negatively associated with mortality (OR 0.61 [0.33–1.12]; *P*=0.11). The results of the multivariable fractional polynomial regression analysis confirm the linear association between heart rate and the probability of myocardial injury. The best-fitting model included one heart rate function that underwent a single linear transformation (Supplementary data, Table S9). A summary plot of this model is shown in Fig. 3, which illustrates that as heart rate increases, the probability of myocardial injury with 30 days of surgery is increased.

## Discussion

The principal finding of this study is that elevated preoperative heart rate is associated with increased incidences of MINS, MI, and death within 30 days of surgery. When we examined the entire range of heart rates, the highest decile (>96 beats min^−1^) was consistently associated with higher incidences of each of these outcomes. Heart rates in the first decile (<60 beats min^−1^) were associated with a lower incidence of mortality, but not MINS or MI. We found similar, but weaker, associations with predefined heart rate thresholds of >70 and >104 beats min^−1^, which were identified from the existing general medical and perioperative literature. The absence of association between heart rates below the 10th decile and outcomes implies that the signal seen with the binary thresholds is attributable mainly to higher event rates in patients with heart rates >96 beats min^−1^. Multivariable fractional polynomial analysis suggests that the relationship between heart rate and myocardial injury is linear.

Our results contrast with those of population-based studies, in which incremental increases in resting heart rate are associated with higher long-term rates of cardiovascular events across the whole heart rate spectrum.^9–12^ If a biological relationship exists between heart rate and clinical outcomes, it may have different profiles in patients undergoing surgery compared with the general population. Therefore, the results of previous perioperative studies using arbitrary predefined heart rate thresholds may not accurately represent the relationship between heart rate and clinical outcomes, whereas the findings of population-based studies are not generalizable to the perioperative period. Our results provoke the question of whether heart rate reduction is a potential therapeutic target to reduce the risk of perioperative cardiovascular events. Previous randomized controlled trials found that mortality and stroke risk was increased by β-blocker therapy.^15^ In our study, 2727 (17.0%) patients received a β-blocker or negatively chronotropic calcium channel blocker within 24 h before surgery. After excluding these patients from the analysis, the independent association between heart rates >96 beats min^−1^ and the outcomes remained. However, the negative association between heart rates <60 beats min^−1^ and mortality was no longer statistically significant, suggesting that the observed association may be confounded by rate-controlling medication. Alternatively, they might suggest that rate-limiting medication was merely a marker of underlying heart disease.

The observed associations between elevated preoperative heart rate and postoperative outcomes offer a plausible mechanism by which tachycardia could promote cardiac complications.^8,29^ The majority of perioperative MIs are thought to originate not from the rupture of atheromatous coronary plaque, as in the general population, but as the result of protracted myocardial ischaemia.^7^ The imbalance between myocardial oxygen supply and demand, and subsequent myocardial ischaemia, is promoted by multiple factors, including anaemia, hypertension, hypotension, and hypoxia.^7^ However, the most prominent candidate mechanism is tachycardia, induced by autonomic imbalance, postoperative pain, hypovolaemia, or reduction in heart rate-limiting medications, leading to increased oxygen consumption and resultant ischaemia.^7,8^ This is supported by evidence from animal studies, where tachycardia induces subendocardial myocardial necrosis.^29^ However, it is possible that tachycardia may simply be a marker of underlying conditions that contribute to myocardial injury, including systemic inflammation and sympathetic autonomic dysfunction.^13,30–32^ Given this uncertainty, it is unclear whether therapeutic control of perioperative heart rate would influence clinical outcome.

This study has several limitations. The influences of premedication and anxiety on heart rate are well established. In an attempt to standardize heart rate measurement, preoperative heart rate was recorded before and as close to induction of anaesthesia as possible. The potential confounding influence of atrial fibrillation, the commonest population-based tachyarrhythmia, was assessed both through the adjustment of the multivariable models and by a sensitivity analysis.^33–35^ Atrial fibrillation was present in only 6% of patients in the top decile, and the removal of these patients had little impact on our findings, nor did exclusion of patients taking β-blockers or rate-limiting calcium channel blockers, or patients undergoing emergency surgery.

The strengths of our analyses derive from the multicentre study design and large patient sample. The sample reflects a wide spectrum of non-cardiac surgery taking place in hospitals in a number of countries, making the results relevant to the majority of surgical patients. The routine measurement of TnT allowed us to identify subclinical myocardial injury in addition to subjective clinical outcomes. We planned the statistical analysis before taking custody of the data and used multivariable models to correct for confounding factors. However, like all observational studies, our results may be susceptible to unmeasured confounding. For example, the highest heart rate decile might include patients in whom the myocardial injury occurred before surgery, something we were unable to account for in our analysis.^36–38^ Nor was the presence of a pacemaker recorded, although it is likely that these patients composed only a small percentage of the study population.

### Conclusion

Elevated preoperative heart rate was associated with MINS, MI, and mortality within 30 days after surgery. This was primarily attributable to significantly higher event rates in patients in the highest decile of heart rate. Further research is needed to understand the effects of heart rate on postoperative myocardial injury and to clarify whether or not heart rate reduction in selected patients can safely reduce major perioperative myocardial ischaemic events.

## Authors’ contributions

Design of the analysis plan: T.E.F.A., G.L.A., R.M.P., P.J.D., R.N.R., R.A.A.

Data analysis: T.E.F.A., R.M.P., G.L.A.

Drafting the manuscript: T.E.F.A., R.M.P., G.L.A.

Revision of the manuscript after critical review: all authors.

## Supplementary material

Supplementary material is available at *British Journal of Anaesthesia* online.

## Declaration of interest

The VISION Study funding sources had no role in the design and conduct of the study; collection, management, analysis, and interpretation of the data; and preparation or approval of the article. R.M.P. has received equipment loans from LiDCO Ltd, a research grant from Circassia Holdings Ltd, and has performed consultancy work for Edwards Lifesciences, Covidien, and Massimo Inc. R.M.P. is a member of the editorial advisory board of the *British Journal of Anaesthesia*. Roche Diagnostics provided the troponin T assays and some financial support for the VISION Study. P.J.D. has received other funding from Roche Diagnostics and Abbott Diagnostics for investigator-initiated studies. All other authors declare they have no conflicts of interest.

## Funding

Funding for this study comes from more than 50 grants for VISION and its sub-studies: Canadian Institutes of Health Research (six grants); Heart and Stroke Foundation of Ontario (two grants); Academic Health Science Centres Alternative Funding Plan Innovation Fund Grant; Population Health Research Institute Grant; Clarity Research Group Grant; McMaster University, Department of Surgery, Surgical Associates Research Grant; Hamilton Health Science New Investigator Fund Grant; Hamilton Health Sciences Grant; Ontario Ministry of Resource and Innovation Grant; Stryker Canada, McMaster University, Department of Anesthesiology (two grants); Saint Joseph′s Healthcare, Department of Medicine (two grants); Father Sean O′Sullivan Research Centre (two grants); McMaster University, Department of Medicine (two grants); Hamilton Health Sciences Summer Studentships (six grants); McMaster University, Department of Clinical Epidemiology and Biostatistics Grant; McMaster University, Division of Cardiology Grant, and Canadian Network and Centre for Trials International Grant; Winnipeg Health Sciences Foundation Operating Grant; Diagnostic Services of Manitoba Research Grant; University of Manitoba, Faculty of Dentistry Operational Fund; Projeto Hospitais de Excelencia a Serviço do SUS grant from the Brazilian Ministry of Health in Partnership with Hcor (Cardiac Hospital Sao Paulo-SP); School of Nursing, Universidad Industrial de Santander; Grupo de Cardiología Preventiva, Universidad Autónoma de Bucaramanga; Fundación Cardioinfantil Instituto de Cardiología; Alianza Diagnóstica SA; University of Malaya Research Grant; and University of Malaya, Penyelidikan Jangka Pendek Grant. Roche Diagnostics provided the troponin T assays and some financial support for the VISION Study. Medical Research Council and *British Journal of Anaesthesia* clinical research training fellowship (grant reference MR/M017974/1 to T.E.F.A.); National Institute for Health Research professorship (to R.P.); *British Journal of Anaesthesia* and Royal College of Anaesthetists basic science fellowship (to G.A.); National Research Foundation of South Africa (to R.N.R.); Heart and Stroke Foundation of Ontario Career Investigator Award (to P.J.D.); Yusuf Chair in Cardiology (P.J.D.).

## Supplementary Material

Supplementary Data
