# Supplementary material for: Preoperative heart rate and myocardial injury after non-cardiac surgery: results of a predefined secondary analysis of the VISION study
Source: Br J Anaesth. 2016 Jul 20;117(2):172–81. doi: 10.1093/bja/aew182 (PMC4954612; doi:10.1093/bja/aew182)
Supplement: Supplementary Data [file supp_117_2_172__index.html]

Supplementary Data 

# Preoperative heart rate and myocardial injury after non-cardiac surgery: results of a predefined secondary analysis of the VISION study

## Supplementary Data

Supplementary Data

- Supplementary Data - Docx file
