# Supplementary material for: Preoperative heart rate and myocardial injury after non-cardiac surgery: results of a predefined secondary analysis of the VISION study
Source: Br J Anaesth. 2016 Jul 20;117(2):172–81. doi: 10.1093/bja/aew182 (PMC4954612; doi:10.1093/bja/aew182)
Supplement: Supplementary Data [file supp_aew182_aew182supp.docx]

**Preoperative heart rate and myocardial injury after non-cardiac surgery: results of the VISION study**

**SUPPLEMENTARY FILE**

**T. E. F. Abbott,^1^ G. L. Ackland,^1^ R. A. Archbold,^2^ A. Wragg,^2^ E. Kam,^1^ T. Ahmad,^1^ A. W. Khan,^3^ E. Niebrzegowska,^2^ R.N. Rodseth,^4^ P. J. Devereaux,^5^ and R.M. Pearse.^1^**

1. *William Harvey Research Institute, Queen Mary University of London, UK*
2. *Barts Health NHS Trust, London UK*
3. *Shaukat Khanum Memorial Cancer Hospital, Lahore, Pakistan*
4. *Nelson R. Mandela School of Medicine, University of KwaZulu-Natal, South Africa*
5. *Population Health Research Institute, McMaster University, Canada*

Correspondence to:

Rupert Pearse

Adult Critical Care Unit

Royal London Hospital

London

E1 1BB

United Kingdom

e-mail: r.pearse@qmul.ac.uk

Tel: +44 20 3594 0351

**Preoperative patient characteristics and types of surgery**

1. Age – Patient age in years

2. Sex – Male or female.

3. Atrial fibrillation – a patient with a current history of atrial fibrillation

4. History of diabetes – Patient states that they have been diagnosed with diabetes or a physician has previously recorded that the patient has diabetes. Includes current gestational, but not past gestational diabetes that has resolved.

5. History of hypertension – A physician diagnosis of hypertension.

6. History of coronary artery disease – A current or prior history of any one of the following: i. angina; ii. myocardial infarction or acute coronary syndrome; iii. a segmental cardiac wall motion abnormality on echocardiography or a segmental fixed defect on radionuclide imaging; iv. a positive radionuclide exercise, echocardiographic exercise, or pharmacological cardiovascular stress test demonstrating cardiac ischemia; v. coronary angiographic or CT coronary angiographic evidence of atherosclerotic stenosis ≥50% of the diameter of any coronary artery; vi. ECG with pathological Q waves in two contiguous leads.

7. History of peripheral vascular disease – A physician diagnosis of a current or prior history of: intermittent claudication, vascular surgery for atherosclerotic disease, an ankle/arm systolic blood pressure ratio ≤ 0.90 in either leg at rest, or angiographic or Doppler study demonstrating ≥ 70% stenosis in a non-cardiac artery.

8. History of stroke or transient ischaemic attack – A physician diagnosis of stroke, CT or MRI evidence of a prior stroke, or physician diagnosis of a prior transient ischaemic attack (TIA).

9. History of chronic obstructive pulmonary disease (COPD) – If the chart or a physician has ever indicated that a patient has chronic bronchitis, accept it has a patient having COPD. If there is no mention of this but the patient tells you they have had daily production of sputum for at least 3 months in 2 consecutive years then they should be marked as having COPD. Likewise if a physician has ever indicated that a patient has emphysema or if a patient’s Pulmonary Function Tests (PFT) state a fixed or irreversible airflow limitation and/or emphysema then they should be marked as having COPD.

10. Preoperative estimated glomerular filtration rate (eGFR) rate as categorical variables (i.e., eGFR <30 ml/minute/1.73m^2^ or receiving dialysis; eGFR of 30 to 44 ml/minute/1.73m^2^; eGFR of 45 to 59 ml/minute/1.73m^2^; and the reference group eGFR of ≥60 ml/minute/1.73m^2^).

11. Urgency of surgery – Emergency surgery was surgery that occurred <24 hours after a patient developed an acute event that led to the need for surgery, and urgent surgery was surgery that occurred 24-72 hours after a patient developed an acute event that led to the need for surgery. Elective surgery was planned greater than 72 hours in advance.

**Ischemic symptoms and signs and electrocardiography findings**

1. Ischemic symptoms/signs included any of the following: chest discomfort, arm discomfort, neck discomfort, jaw discomfort, shortness of breath, or pulmonary oedema. These ischemic symptoms or signs had to have occurred within 24 hours of an elevated troponin measurement.

2. Ischemic electrocardiography findings included any of the following:

i. development of pathologic Q waves in any two contiguous leads that were ≥30 milliseconds; ii. development of left bundle branch block (LBBB); or

iii. development of ST segment elevation (≥2 mm in leads V_1_, V_2_, or V_3_ OR ≥1 mm in the other leads), ST segment depression (≥1 mm), or symmetric inversion of T waves ≥1 mm in at least two contiguous leads.

ST segment elevation, ST segment depression, and LBBB had to have occurred within 3 days of an elevated troponin measurement, and symmetric T wave inversion had to have occurred within 5 days of an elevated troponin measurement.

**Supplementary table 1. Multivariable logistic regression models for preoperative heart rate deciles**

**Sensitivity analysis using heart rate 60-64bpm as the reference category. Dependent variables are myocardial injury, myocardial infarction and mortality within 30 days of surgery. Preoperative heart rate was stratified by decile. Each decile was compared to the second decile of heart rate (60-64bpm).**

|  | Myocardial Injury | | Myocardial Infarction | | Mortality | |
| --- | --- | --- | --- | --- | --- | --- |
| Covariates | odds ratio | p-value | odds ratio | p-value | odds ratio | p-value |
| Age (years) |  |  |  |  |  |  |
| 45-64 (reference) | - | - | - | - | - | - |
| 65-75 | 1.08 (0.90-1.30) | 0.43 | 1.16 (0.87-1.55) | 0.30 | 1.64 (1.17-2.30) | <0.01 |
| >75 | 2.08 (1.74-2.48) | <0.01 | 1.90 (1.45-2.49) | <0.01 | 2.41 (1.73-3.35) | <0.01 |
| Male sex | 1.40 (1.22-1.61) | <0.01 | 1.04 (0.85-1.28) | 0.70 | 1.27 (0.99-1.63) | 0.06 |
| History of AF | 1.53 (1.18-2.00) | <0.01 | 1.29 (0.90-1.85) | 0.17 | 0.97 (0.60-1.56) | 0.89 |
| History of diabetes | 1.39 (1.19-1.61) | <0.01 | 1.21 (0.97-1.52) | 0.10 | 0.98 (0.73-1.32) | 0.91 |
| History of hypertension | 1.31 (1.12-1.54) | <0.01 | 1.41 (1.10-1.80) | 0.01 | 0.98 (0.75-1.29) | 0.89 |
| History of heart failure | 1.59 (1.26-1.99) | <0.01 | 1.67 (1.24-2.25) | <0.01 | 1.38 (0.92-2.10) | 0.12 |
| History of coronary artery disease | 1.48 (1.25-1.76) | <0.01 | 2.23 (1.77-2.81) | <0.01 | 0.96 (0.68-1.34) | 0.80 |
| History of peripheral vascular disease | 2.17 (1.77-2.65) | <0.01 | 2.11 (1.60-2.78) | <0.01 | 1.75 (1.21-2.53) | <0.01 |
| History of stroke or transient ischaemic attack | 1.46 (1.20-1.78) | <0.01 | 1.14 (0.85-1.52) | 0.37 | 1.53 (1.10-2.15) | 0.01 |
| Preoperative eGFR (ml/min) |  |  |  |  |  |  |
| <30 | 10.75 (8.69-13.29) | <0.01 | 3.98 (2.96-5.36) | <0.01 | 2.95 (2.01-4.31) | <0.01 |
| 30-45 | 2.51 (2.02-3.12) | <0.01 | 1.69 (1.22-2.34) | <0.01 | 1.58 (1.07-2.36) | 0.02 |
| 45-60 | 1.68 (1.39-2.03) | <0.01 | 1.40 (1.04-1.87) | 0.02 | 0.94 (0.63-1.40) | 0.77 |
| >60 (reference) | - | - | - | - | - | - |
| History of chronic obstructive pulmonary disease | 1.18 (0.97-1.45) | 0.10 | 1.12 (0.83-1.49) | 0.46 | 1.93 (1.40-2.65) | <0.01 |
| Neurosurgery | 1.14 (0.87-1.51) | 0.34 | 0.58 (0.35-0.98) | 0.04 | 1.82 (1.17-2.82) | <0.01 |
| Urgent or emergency surgery | 1.82 (1.54-2.15) | <0.01 | 2.14 (1.70-2.69) | <0.01 | 3.11 (2.41-4.02) | <0.01 |
| Major Surgery | 1.66 (1.42-1.93) | <0.01 | 2.19 (1.71-2.80) | <0.01 | 1.51 (1.13-2.02) | <0.01 |
| Preoperative heart rate (beats per minute) |  |  |  |  |  |  |
| <60 | 1.08 (0.79-1.48) | 0.63 | 1.23 (0.74-2.05) | 0.42 | 0.71 (0.33-1.54) | 0.39 |
| 60-64 (reference) | - | - | - | - | - | - |
| 65-68 | 1.03 (0.75-1.42) | 0.84 | 1.38 (0.84-2.26) | 0.20 | 0.86 (0.41-1.81) | 0.69 |
| 69-71 | 1.18 (0.86-1.63) | 0.30 | 1.68 (1.03-2.73) | 0.04 | 1.51 (0.78-2.93) | 0.22 |
| 72-74 | 0.83 (0.58-1.17) | 0.28 | 1.03 (0.59-1.80) | 0.91 | 0.99 (0.47-2.08) | 0.98 |
| 75-79 | 1.03 (0.77-1.39) | 0.83 | 1.45 (0.91-2.30) | 0.12 | 1.58 (0.85-2.91) | 0.15 |
| 80-82 | 1.19 (0.87-1.61) | 0.28 | 1.35 (0.83-2.21) | 0.23 | 1.57 (0.83-2.95) | 0.17 |
| 83-87 | 1.59 (1.17-2.16) | <0.01 | 1.57 (0.95-2.58) | 0.08 | 1.49 (0.77-2.91) | 0.24 |
| 88-96 | 1.30 (0.98-1.73) | 0.07 | 1.28 (0.80-2.05) | 0.30 | 2.06 (1.15-3.68) | 0.02 |
| >96 | 1.73 (1.29-2.31) | <0.01 | 2.38 (1.52-3.71) | <0.01 | 2.24 (2.55-7.75) | <0.01 |
|  |  |  |  |  |  |  |

**Supplementary table 2. Multivariable logistic regression models for preoperative heart rate deciles – excluding atrial fibrillation cases.**

**Sensitivity analysis without AF. Dependent variables are myocardial injury, myocardial infarction and mortality within 30 days of surgery. Preoperative heart rate was stratified by decile. Each decile was compared to the unweighted average heart rate for the whole sample.**

|  | Myocardial Injury | | Myocardial Infarction | | Mortality | |
| --- | --- | --- | --- | --- | --- | --- |
| Covariates | odds ratio | p-value | odds ratio | p-value | odds ratio | p-value |
| Age (years) |  |  |  |  |  |  |
| 45-64 (reference) | - | - | - | - | - | - |
| 65-75 | 1.08 (0.90-1.31) | 0.42 | 1.20 (0.90-1.61) | 0.22 | 1.71 (1.21-2.40) | <0.01 |
| >75 | 2.08 (1.74-2.50) | <0.01 | 1.93 (1.45-2.55) | <0.01 | 2.44 (1.74-3.43) | <0.01 |
| Male sex | 1.38 (1.20-1.60) | <0.01 | 0.99 (0.80-1.24) | 0.95 | 1.26 (0.98-1.64) | 0.08 |
| History of diabetes | 1.44 (1.23-1.69) | <0.01 | 1.26 (0.99-1.59) | 0.06 | 0.99 (0.73-1.35) | 0.95 |
| History of hypertension | 1.27 (1.08-1.50) | <0.01 | 1.37 (1.06-1.78) | 0.02 | 0.91 (0.69-1.22) | 0.54 |
| History of heart failure | 1.70 (1.32-2.19) | <0.01 | 1.74 (1.25-2.42) | <0.01 | 1.24 (0.76-2.00) | 0.39 |
| History of coronary artery disease | 1.61 (1.35-1.92) | <0.01 | 2.47 (1.93-3.16) | <0.01 | 1.00 (0.70-1.44) | 0.99 |
| History of peripheral vascular disease | 2.09 (1.68-2.59) | <0.01 | 2.02 (1.50-2.73) | <0.01 | 1.68 (1.13-2.51) | 0.01 |
| History of stroke or transient ischaemic attack | 1.48 (1.20-1.83) | <0.01 | 1.01 (0.73-1.40) | 0.94 | 1.55 (1.08-2.23) | 0.02 |
| Preoperative eGFR (ml/min) |  |  |  |  |  |  |
| <30 | 10.40 (8.33-12.98) | <0.01 | 3.91 (2.84-5.38) | <0.01 | 2.31 (2.22-4.91) | <0.01 |
| 30-45 | 2.49 (1.98-3.13) | <0.01 | 1.71 (1.21-2.42) | <0.01 | 1.53 (0.99-2.37) | 0.05 |
| 45-60 | 1.70 (1.39-2.07) | <0.01 | 1.49 (1.10-2.02) | 0.01 | 1.01 (0.67-1.52) | 0.96 |
| >60 (reference) | - | - | - | - | - | - |
| History of chronic obstructive pulmonary disease | 1.20 (0.97-1.48) | 0.10 | 1.24 (0.92-1.69) | 0.16 | 2.12 (1.53-2.95) | <0.01 |
| Neurosurgery | 1.19 (0.90-1.58) | 0.22 | 0.59 (0.35-1.02) | 0.06 | 1.92 (1.23-2.98) | <0.01 |
| Urgent or emergency surgery | 1.94 (1.64-2.30) | <0.01 | 2.23 (1.75-2.84) | <0.01 | 3.21 (2.46-4.19) | <0.01 |
| Major Surgery | 1.62 (1.38-1.91) | <0.01 | 2.15 (1.65-2.79) | <0.01 | 1.51 (1.12-2.05) | 0.01 |
| Preoperative heart rate (beats per minute) |  |  |  |  |  |  |
| <60 | 0.90 (0.71-1.11) | 0.30 | 0.87 (0.61-1.23) | 0.43 | 0.53 (0.30-0.93) | 0.03 |
| 60-64 | 0.82 (0.66-1.03) | 0.09 | 0.69 (0.48-0.99) | 0.05 | 0.71 (0.44-1.15) | 0.16 |
| 65-68 | 0.90 (0.72-1.12) | 0.33 | 1.05 (0.76-1.45) | 0.77 | 0.60 (0.35-1.02) | 0.06 |
| 69-71 | 1.01 (0.81-1.27) | 0.92 | 1.14 (0.82-1.58) | 0.44 | 1.03 (0.67-1.59) | 0.89 |
| 72-74 | 0.73 (0.56-0.94) | 0.01 | 0.79 (0.54-1.17) | 0.24 | 0.73 (0.44-1.23) | 0.24 |
| 75-79 | 0.89 (0.73-1.08) | 0.24 | 1.05 (0.79-1.40) | 0.72 | 1.17 (0.82-1.66) | 0.40 |
| 80-82 | 0.95 (0.77-1.18) | 0.64 | 0.88 (0.62-1.23) | 0.45 | 1.04 (0.70-1.56) | 0.85 |
| 83-87 | 1.39 (1.13-1.71) | <0.01 | 1.13 (0.81-1.58) | 0.46 | 1.09 (0.71-1.67) | 0.70 |
| 88-96 | 1.12 (0.94-1.35) | 0.22 | 0.90 (0.67-1.21) | 0.48 | 1.41 (1.03-1.93) | 0.03 |
| >96 | 1.57 (1.30-1.90) | <0.01 | 1.87 (1.45-2.42) | <0.01 | 3.18 (2.44-4.15) | <0.01 |
|  |  |  |  |  |  |  |

**Supplementary table 3. Multivariable logistic regression models for preoperative heart rate deciles – excluding emergency cases.**

**Sensitivity analysis without emergencies. Dependent variables are myocardial injury, myocardial infarction and mortality within 30 days of surgery. Preoperative heart rate was stratified by decile. Each decile was compared to the unweighted average heart rate for the whole sample.**

|  | Myocardial Injury | | Myocardial Infarction | | Mortality | |
| --- | --- | --- | --- | --- | --- | --- |
| Covariates | odds ratio | p-value | odds ratio | p-value | odds ratio | p-value |
| Age (years) |  |  |  |  |  |  |
| 45-64 (reference) | - | - | - | - | - | - |
| 65-75 | 1.09 (0.89-1.33) | 0.39 | 1.20 (0.87-1.66) | 0.26 | 1.71 (1.16-2.52) | 0.01 |
| >75 | 2.10 (1.73-2.56) | <0.01 | 2.26 (1.67-3.07) | <0.01 | 2.58 (1.74-3.83) | <0.01 |
| Male sex | 1.42 (1.21-1.65) | <0.01 | 1.07 (0.85-1.35) | 0.58 | 1.34 (0.99-1.80) | 0.06 |
| History of AF | 1.88 (1.42-2.48) | <0.01 | 1.48 (1.00-2.21) | 0.05 | 1.13 (0.65-1.97) | 0.66 |
| History of diabetes | 1.33 (1.12-1.58) | <0.01 | 1.15 (0.88-1.49) | 0.30 | 0.85 (0.59-1.23) | 0.40 |
| History of hypertension | 1.24 (1.04-1.48) | 0.02 | 1.26 (0.95-1.67) | 0.10 | 1.03 (0.74-1.43) | 0.87 |
| History of heart failure | 1.46 (1.14-1.89) | <0.01 | 1.44 (1.01-2.04) | 0.04 | 1.13 (0.67-1.91) | 0.65 |
| History of coronary artery disease | 1.56 (1.30-1.88) | <0.01 | 2.41 (1.85-3.13) | <0.01 | 1.11 (0.75-1.64) | 0.61 |
| History of peripheral vascular disease | 2.03 (1.62-2.55) | <0.01 | 2.12 (1.55-2.90) | <0.01 | 1.45 (0.91-2.29) | 0.12 |
| History of stroke or transient ischaemic attack | 1.50 (1.21-1.87) | <0.01 | 1.14 (0.82-1.59) | 0.42 | 1.88 (1.28-2.77) | <0.01 |
| Preoperative eGFR (ml/min) |  |  |  |  |  |  |
| <30 | 11.80 (9.32-14.93) | <0.01 | 4.07 (2.87-5.77) | <0.01 | 2.22 (1.33-3.71) | <0.01 |
| 30-45 | 2.71 (2.13-3.44) | <0.01 | 1.73 (1.20-2.51) | <0.01 | 1.54 (0.95-2.49) | 0.08 |
| 45-60 | 1.65 (1.33-2.04) | <0.01 | 1.38 (1.00-1.93) | 0.05 | 0.78 (0.47-1.27) | 0.32 |
| >60 (reference) | - | - | - | - | - | - |
| History of chronic obstructive pulmonary disease | 1.24 (1.00-1.54) | 0.05 | 1.09 (0.79-1.52) | 0.59 | 2.19 (1.52-3.14) | <0.01 |
| Neurosurgery | 1.13 (0.84-1.52) | 0.41 | 0.57 (0.33-1.00) | 0.05 | 1.98 (1.22-3.21) | 0.01 |
| Urgent or emergency surgery | 1.94 (1.40-2.68) | <0.01 | 2.28 (1.49-3.51) | <0.01 | 3.47 (2.26-5.32) | <0.01 |
| Major Surgery | 1.68 (1.42-1.99) | <0.01 | 2.44 (1.84-3.24) | <0.01 | 1.23 (0.89-1.71) | 0.21 |
| Preoperative heart rate (beats per minute) |  |  |  |  |  |  |
| <60 | 0.88 (0.70-1.10) | 0.25 | 0.79 (0.55-1.14) | 0.21 | 0.39 (0.19-0.77) | 0.01 |
| 60-64 | 0.83 (0.67-1.04) | 0.11 | 0.70 (0.48-1.02) | 0.07 | 0.77 (0.47-1.28) | 0.31 |
| 65-68 | 0.87 (0.69-1.09) | 0.22 | 0.95 (0.67-1.35) | 0.78 | 0.62 (0.34-1.11) | 0.11 |
| 69-71 | 1.08 (0.86-1.36) | 0.51 | 1.37 (1.00-1.89( | 0.05 | 1.15 (0.72-1.84) | 0.55 |
| 72-74 | 0.73 (0.56-0.95) | 0.02 | 0.81 (0.53-1.22) | 0.31 | 0.60 (0.32-1.15) | 0.13 |
| 75-79 | 0.84 (0.68-1.04) | 0.10 | 0.96 (0.70-1.32) | 0.81 | 1.26 (0.85-1.87) | 0.26 |
| 80-82 | 1.04 (0.83-1.29) | 0.76 | 1.04 (0.74-1.47) | 0.82 | 1.42 (0.93-2.15) | 0.10 |
| 83-87 | 1.33 (1.06-1.66) | 0.01 | 0.99 (0.68-1.46) | 0.98 | 0.83 (0.47-1.45) | 0.51 |
| 88-96 | 1.15 (0.95-1.40) | 0.16 | 1.00 (0.73-1.38) | 0.98 | 1.58 (1.10-2.27) | 0.01 |
| >96 | 1.52 (1.24-1.87) | <0.01 | 1.71 (1.27-2.30) | <0.01 | 3.38 (2.47-4.62) | <0.01 |
|  |  |  |  |  |  |  |

**Supplementary table 4. Preoperative heart rate threshold of 70 beats per minute – excluding atrial fibrillation cases.**

**Sensitivity analysis without AF cases. Multivariable logistic regression models for myocardial injury, myocardial infarction and mortality, all within 30-days of non-cardiac surgery. Preoperative heart rate was stratified according to a threshold of 70 beats per minute.**

|  | Myocardial Injury | | Myocardial Infarction | | Mortality | |
| --- | --- | --- | --- | --- | --- | --- |
| Covariates | odds ratio | p-value | odds ratio | p-value | odds ratio | p-value |
| Age (years) |  |  |  |  |  |  |
| 45-64 (reference) | - | - | - | - | - | - |
| 65-75 | 1.07 (0.89-1.30) | 0.48 | 1.18 (0.88-1.59) | 0.26 | 1.65 (1.17-2.32) | <0.01 |
| >75 | 2.03 (1.69-2.43) | <0.01 | 1.87 (1.41-2.48) | <0.01 | 2.29 (1.63-3.22) | <0.01 |
| Male sex | 1.37 (1.18-1.58) | <0.01 | 0.98 (0.79-1.22) | 0.87 | 1.26 (0.98-1.63) | 0.08 |
| History of diabetes | 1.45 (1.24-1.70) | <0.01 | 1.28 (1.01-1.62) | 0.05 | 1.01 (0.74-1.36) | 0.98 |
| History of hypertension | 1.27 (1.08-1.50) | <0.01 | 1.36 (1.05-1.76) | 0.02 | 0.91 (0.69-1.22) | 0.54 |
| History of heart failure | 1.70 (1.32-2.19) | <0.01 | 1.73 (1.25-2.41) | <0.01 | 1.24 (0.77-2.00) | 0.38 |
| History of coronary artery disease | 1.59 (1.33-1.90) | <0.01 | 2.42 (1.90-3.09) | <0.01 | 0.97 (0.68-1.39) | 0.89 |
| History of peripheral vascular disease | 2.07 (1.67-2.56) | <0.01 | 2.02 (1.49-2.72) | <0.01 | 1.70 (1.14-2.52) | <0.01 |
| History of stroke or transient ischaemic attack | 1.48 (1.20-1.83) | <0.01 | 1.01 (0.73-1.39) | 0.96 | 1.56 (1.09-2.24) | 0.02 |
| Preoperative eGFR (ml/min) |  |  |  |  |  |  |
| <30 | 10.57 (8.48-13.17) | <0.01 | 4.03 (2.94-5.54) | <0.01 | 3.63 (2.45-5.37) | <0.01 |
| 30-45 | 2.50 (1.99-3.14) | <0.01 | 1.76 (1.25-2.49) | <0.01 | 1.64 (1.06-2.52) | 0.03 |
| 45-60 | 1.72 (1.40-2.09) | <0.01 | 1.50 (1.11-2.04) | <0.01 | 1.04 (0.69-1.56) | 0.86 |
| >60 (reference) | - | - | - | - | - | - |
| History of chronic obstructive pulmonary disease | 1.22 (0.98-1.50) | 0.07 | 1.27 (0.94-1.72) | 0.12 | 2.17 (1.57-3.01) | <0.01 |
| Neurosurgery | 1.19 (0.90-1.58) | 0.22 | 0.60 (0.35-1.02) | 0.06 | 1.90 (1.23-2.95) | <0.01 |
| Urgent or emergency surgery | 2.05 (1.73-2.42) | <0.01 | 2.41 (1.90-3.06) | <0.01 | 3.62 (2.79-4.69) | <0.01 |
| Major Surgery | 1.63 (1.39-1.92) | <0.01 | 2.17 (1.67-2.82) | <0.01 | 1.56 (1.15-2.12) | <0.01 |
| Heart rate threshold (beats per minute) |  |  |  |  |  |  |
| >70 | 1.11 (1.03-1.19) | <0.01 | 1.08 (0.96-1.21) | 0.21 | 1.53 (1.30-1.80) | <0.01 |
|  |  |  |  |  |  |  |

**Supplementary table 5. Preoperative heart rate threshold of 104 beats per minute – excluding atrial fibrillation cases.**

**Sensitivity analysis excluding AF cases. Multivariable logistic regression models for myocardial injury, myocardial infarction and mortality, all within 30-days of non-cardiac surgery. Preoperative heart rate was stratified according to a threshold of 104 beats per minute.**

|  | Myocardial Injury | | Myocardial Infarction | | Mortality | |
| --- | --- | --- | --- | --- | --- | --- |
| Covariates | odds ratio | p-value | odds ratio | p-value | odds ratio | p-value |
| Age (years) |  |  |  |  |  |  |
| 45-64 (reference) | - | - | - | - | - | - |
| 65-75 | 1.08 (0.89-1.30) | 0.45 | 1.20 (0.89-1.60) | 0.24 | 1.67 (1.19-2.35) | <0.01 |
| >75 | 2.06 (1.72-2.47) | <0.01 | 1.91 (1.45-2.54) | <0.01 | 2.41 (1.72-3.39) | <0.01 |
| Male sex | 1.35 (1.17-1.56) | <0.01 | 0.98 (0.79-1.21) | 0.82 | 1.21 (0.93-1.56) | 0.15 |
| History of diabetes | 1.46 (1.24-1.71) | <0.01 | 1.27 (1.01-1.61) | 0.05 | 1.02 (0.75-1.38) | 0.92 |
| History of hypertension | 1.28 (1.09-1.51) | <0.01 | 1.37 (1.05-1.77) | 0.02 | 0.93 (0.70-1.23) | 0.60 |
| History of heart failure | 1.71 (1.33-2.20) | <0.01 | 1.75 (1.26-2.44) | <0.01 | 1.25 (0.77-2.02) | 0.36 |
| History of coronary artery disease | 1.56 (1.31-1.86) | <0.01 | 2.40 (1.88-3.06) | <0.01 | 0.90 (0.63-1.29) | 0.56 |
| History of peripheral vascular disease | 1.08 (1.68-2.57) | <0.01 | 2.02 (1.50-2.73) | <0.01 | 1.70 (1.14-2.52) | <0.01 |
| History of stroke or transient ischaemic attack | 1.47 (1.20-1.82) | <0.01 | 1.01 (0.73-1.40) | 0.95 | 1.56 (1.09-2.23) | 0.02 |
| Preoperative eGFR (ml/min) |  |  |  |  |  |  |
| <30 | 10.39 (8.33-12.95) | <0.01 | 3.91 (2.84-5.37) | <0.01 | 3.38 (2.27-5.03) | <0.01 |
| 30-45 | 2.47 (1.96-3.11) | <0.01 | 1.72 (1.22-2.43) | <0.01 | 1.50 (0.98-2.32) | 0.07 |
| 45-60 | 1.69 (1.39-2.07) | <0.01 | 1.47 (1.10-2.01) | 0.01 | 1.00 (0.67-1.51) | 0.99 |
| >60 (reference) | - | - | - | - | - | - |
| History of chronic obstructive pulmonary disease | 1.22 (0.99-1.51) | 0.07 | 1.26 (0.93-1.71) | 0.13 | 2.22 (1.60-3.08) | <0.01 |
| Neurosurgery | 1.19 (0.90-1.57) | 0.23 | 0.60 (0.35-1.03) | 0.06 | 1.94 (1.19-2.85) | <0.01 |
| Urgent or emergency surgery | 2.02 (1.70-2.39) | <0.01 | 2.33 (1.83-2.96) | <0.01 | 3.58 (2.76-4.66) | <0.01 |
| Major Surgery | 1.64 (1.39-1.93) | <0.01 | 2.16 (1.66-2.80) | <0.01 | 1.57 (1.16-2.13) | <0.01 |
| Heart rate threshold (beats per minute) |  |  |  |  |  |  |
| >104 | 1.42 (1.24-1.63) | <0.01 | 1.39 (1.15-1.68) | <0.01 | 1.92 (1.61-2.30) | <0.01 |
|  |  |  |  |  |  |  |

**Supplementary table 6. Preoperative heart rate threshold of 70 beats per minute – excluding emergency cases.**

**Sensitivity analysis without emergency cases. Multivariable logistic regression models for myocardial injury, myocardial infarction and mortality, all within 30-days of non-cardiac surgery. Preoperative heart rate was stratified according to a threshold of 70 beats per minute.**

|  | Myocardial Injury | | Myocardial Infarction | | Mortality | |
| --- | --- | --- | --- | --- | --- | --- |
| Covariates | odds ratio | p-value | odds ratio | p-value | odds ratio | p-value |
| Age (years) |  |  |  |  |  |  |
| 45-64 (reference) | - | - | - | - | - | - |
| 65-75 | 1.08 (0.88-1.32) | 0.46 | 1.19 (0.86-1.64) | 0.29 | 1.66 (1.13-2.44) | 0.01 |
| >75 | 2.04 (1.68-2.48) | <0.01 | 2.20 (1.62-2.98) | <0.01 | 2.45 (1.66-3.63) | <0.01 |
| Male sex | 1.40 (1.20-1.63) | <0.01 | 1.05 (0.83-1.32) | 0.72 | 1.31 (0.98-1.77) | 0.07 |
| History of atrial fibrillation | 1.97 (1.49-2.60) | <0.01 | 1.56 (1.05-2.33) | 0.03 | 1.26 (0.72-2.18) | 0.42 |
| History of diabetes | 1.34 (1.13-1.59) | <0.01 | 1.17 (0.90-1.52) | 0.23 | 0.87 (0.61-1.25) | 0.45 |
| History of hypertension | 1.24 (1.04-1.48) | 0.02 | 1.24 (0.94-1.64) | 0.13 | 1.03 (0.74-1.43) | 0.86 |
| History of heart failure | 1.45 (1.13-1.86) | <0.01 | 1.43 (1.01-2.03) | 0.04 | 1.08 (0.64-1.83) | 0.77 |
| History of coronary artery disease | 1.54 (1.28-1.85) | <0.01 | 2.36 (1.82-3.06) | <0.01 | 1.10 (0.75-1.63) | 0.62 |
| History of peripheral vascular disease | 2.02 (1.61-2.53) | <0.01 | 2.12 (1.55-2.90) | <0.01 | 1.47 (0.93-2.34) | 0.10 |
| History of stroke or transient ischaemic attack | 1.51 (1.22-1.88) | <0.01 | 1.16 (0.84-1.62) | 0.36 | 1.91 (1.30-2.80) | <0.01 |
| Preoperative eGFR (ml/min) |  |  |  |  |  |  |
| <30 | 11.86 (9.38-15.01) | <0.01 | 4.13 (2.92-5.86) | <0.01 | 2.29 (1.37-3.82) | <0.01 |
| 30-45 | 2.72 (2.14-3.46) | <0.01 | 1.74 (1.20-2.52) | <0.01 | 1.60 (0.99-2.59) | 0.05 |
| 45-60 | 1.66 (1.34-2.06) | <0.01 | 1.38 (0.99-1.93) | 0.05 | 0.80 (0.49-1.31) | 0.38 |
| >60 (reference) | - | - | - | - | - | - |
| History of chronic obstructive pulmonary disease | 1.26 (1.02-1.57) | 0.04 | 1.11 (0.80-1.54) | 0.53 | 2.21 (1.54-3.18) | <0.01 |
| Neurosurgery | 1.13 (0.84-1.51) | 0.43 | 0.56 (0.32-0.99) | 0.04 | 1.93 (1.19-3.12) | <0.01 |
| Urgent surgery | 2.11 (1.53-2.92) | <0.01 | 2.58 (1.70-3.93) | <0.01 | 4.19 (2.76-6.35) | <0.01 |
| Major Surgery | 1.69 (1.43-2.00) | <0.01 | 2.46 (1.85-3.26) | <0.01 | 1.26 (0.91-1.74) | 0.17 |
| Heart rate threshold (beats per minute) |  |  |  |  |  |  |
| >70 | 1.09 (1.01-1.18) | 0.02 | 1.06 (0.94-1.20) | 0.33 | 1.57 (1.32-1.88) | <0.01 |
|  |  |  |  |  |  |  |

**Supplementary table 7. Preoperative heart rate threshold of 104 beats per minute – excluding emergency cases.**

**Sensitivity analysis excluding emergency cases. Multivariable logistic regression models for myocardial injury, myocardial infarction and mortality, all within 30-days of non-cardiac surgery. Preoperative heart rate was stratified according to a threshold of 104 beats per minute.**

|  | Myocardial Injury | | Myocardial Infarction | | Mortality | |
| --- | --- | --- | --- | --- | --- | --- |
| Covariates | odds ratio | p-value | odds ratio | p-value | odds ratio | p-value |
| Age (years) |  |  |  |  |  |  |
| 45-64 (reference) | - | - | - | - | - | - |
| 65-75 | 1.08 (0.89-1.32) | 0.45 | 1.19 (0.87-1.64) | 0.28 | 1.65 (1.12-2.43) | 0.01 |
| >75 | 2.06 (1.70-2.50) | <0.01 | 2.23 (1.65-3.03) | <0.01 | 2.47 (1.67-3.66) | <0.01 |
| Male sex | 1.39 (1.19-1.62) | <0.01 | 1.04 (0.82-1.31) | 0.75 | 1.24 (0.92-1.67) | 0.15 |
| History of atrial fibrillation | 1.86 (1.40-2.47) | <0.01 | 1.48 (0.99-2.20) | 0.06 | 1.16 (0.67-2.03) | 0.60 |
| History of diabetes | 1.35 (1.14-1.60) | <0.01 | 1.18 (0.91-1.53) | 0.21 | 0.90 (0.62-1.29) | 0.55 |
| History of hypertension | 1.25 (1.05-1.49) | 0.01 | 1.24 (0.94-1.64) | 0.13 | 1.03 (0.74-1.43) | 0.86 |
| History of heart failure | 1.48 (1.15-1.90) | <0.01 | 1.47 (1.04-2.08) | 0.03 | 1.13 (0.67-1.90) | 0.66 |
| History of coronary artery disease | 1.53 (1.27-1.84) | <0.01 | 2.36 (1.82-3.06) | <0.01 | 1.02 (0.69-1.50) | 0.94 |
| History of peripheral vascular disease | 2.01 (1.61-2.52) | <0.01 | 2.12 (1.55-2.89) | <0.01 | 1.46 (0.92-2.32) | 0.10 |
| History of stroke or transient ischaemic attack | 1.50 (1.21-1.86) | <0.01 | 1.15 (0.83-1.59) | 0.41 | 1.84 (1/25-2.71) | <0.01 |
| Preoperative eGFR (ml/min) |  |  |  |  |  |  |
| <30 | 11.78 (9.31-14.90) | <0.01 | 4.07 (2.87-5.77) | <0.01 | 2.23 (1.33-3.74) | <0.01 |
| 30-45 | 2.69 (2.12-3.42) | <0.01 | 1.70 (1.17-2.47) | <0.01 | 1.51 (0.93-2.45) | 0.10 |
| 45-60 | 1.65 (1.33-2.04) | <0.01 | 1.38 (0.99-1.91) | 0.06 | 0.77 (0.47-1.26) | 0.30 |
| >60 (reference) | - | - | - | - | - | - |
| History of chronic obstructive pulmonary disease | 1.27 (1.02-1.57) | 0.03 | 1.11 (0.80-1.54) | 0.53 | 2.32 (1.62-3.33) | <0.01 |
| Neurosurgery | 1.13 (0.84-1.51) | 0.42 | 0.57 (0.33-1.00) | 0.05 | 1.83 (1.13-2.96) | 0.01 |
| Urgent surgery | 2.01 (1.45-2.78) | <0.01 | 2.33 (1.52-3.58) | <0.01 | 3.95 (2.58-6.04) | <0.01 |
| Major Surgery | 1.70 (1.44-2.02) | <0.01 | 2.46 (1.85-3.26) | <0.01 | 1.29 (0.93-1.78) | 0.13 |
| Heart rate threshold (beats per minute) |  |  |  |  |  |  |
| ≤104 | 0.68 (0.59-0.79) | <0.01 | 0.69 (0.56-0.85) | <0.01 | 0.53 (0.43-0.66) | <0.01 |
| >104 | 1.46 (1.26-1.70) | <0.01 | 1.44 (1.17-1.78) | <0.01 | 1.88 (1.52-2.33) | <0.01 |
|  |  |  |  |  |  |  |

**Supplementary table 8. Multivariable logistic regression models for preoperative heart rate deciles – excluding rate limiting medication**

**Sensitivity analysis excluding beta-blockers and rate limiting calcium channel blockers. Multivariable logistic regression models for myocardial injury, myocardial infarction and mortality, all within 30-days of non-cardiac surgery.**

|  | Myocardial Injury | | Myocardial Infarction | | Mortality | |
| --- | --- | --- | --- | --- | --- | --- |
| Covariates | odds ratio | p-value | odds ratio | p-value | odds ratio | p-value |
| Age (years) |  |  |  |  |  |  |
| 45-64 (reference) | - | - | - | - | - | - |
| 65-75 | 1.01 (0.88-1.36) | 0.41 | 1.08 (0.76-1.53) | 0.67 | 1.70 (1.18-2.44) | <0.01 |
| >75 | 2.28 (1.86-2.81) | <0.01 | 1.88 (1.35-2.60) | <0.01 | 2.34 (1.63-3.35) | <0.01 |
| Male sex | 1.27 (1.07-1.49) | <0.01 | 0.80 (0.62-1.03) | <0.01 | 1.20 (0.91-1.57) | 0.20 |
| History of AF | 1.75 (1.22-2.51) | <0.01 | 1.29 (0.78-2.13) | 0.33 | 0.82 (0.42-1.58) | 0.55 |
| History of diabetes | 1.31 (1.08-1.58) | <0.01 | 1.29 (0.97-1.72) | 0.08 | 0.99 (0.71-1.38) | 0.94 |
| History of hypertension | 1.19 (0.99-1.42) | 0.06 | 1.20 (0.90-1.59) | 0.21 | 0.99 (0.74-1.33) | 0.94 |
| History of heart failure | 1.76 (1.30-2.38) | <0.01 | 2.09 (1.42-3.08) | <0.01 | 1.23 (0.72-2.09) | 0.45 |
| History of coronary artery disease | 1.56 (1.24-1.97) | <0.01 | 2.37 (1.74-3.23) | <0.01 | 1.02 (0.67-1.54) | 0.94 |
| History of peripheral vascular disease | 1.85 (1.42-2.40) | <0.01 | 2.01(1.40-2.90) | <0.01 | 1.86 (1.22-2.82) | <0.01 |
| History of stroke or transient ischaemic attack | 1.52 (1.19-1.94) | <0.01 | 1.15 (0.79-1.68) | 0.47 | 1.67 (1.14-2.44) | 0.01 |
| Preoperative eGFR (ml/min) |  |  |  |  |  |  |
| <30 | 12.83 (9.90-16.61) | <0.01 | 3.95 (2.70-5.80) | <0.01 | 3.34 (2.16-5.15) | <0.01 |
| 30-45 | 2.90 (2.24-3.77) | <0.01 | 1.94 (1.30-2.90) | <0.01 | 1.85 (1.19-2.89) | <0.01 |
| 45-60 | 1.72 (1.36-2.16) | <0.01 | 1.56 (1.09-2.24) | 0.01 | 1.04 (0.67-1.60) | 0.87 |
| >60 (reference) | - | - | - | - | - | - |
| History of chronic obstructive pulmonary disease | 1.36 (1.07-1.71) | 0.01 | 1.53 (1.09-2.15) | 0.01 | 2.24 (1.59-3.16) | <0.01 |
| Neurosurgery | 1.23 (0.89-1.69) | 0.21 | 0.65 (0.34-1.21) | 0.17 | 1.99 (1.25-3.17) | <0.01 |
| Urgent or emergency surgery | 1.81 (1.49-2.18) | <0.01 | 2.35 (1.80-3.08) | <0.01 | 3.32 (2.51-4.38) | <0.01 |
| Major Surgery | 1.74 (1.45-2.09) | <0.01 | 2.27 (1.68-3.09) | <0.01 | 1.63 (1.18-2.25) | <0.01 |
| Preoperative heart rate (beats per minute) |  |  |  |  |  |  |
| <60 | 0.92 (0.70-1.21) | 0.56 | 0.81 (0.50-1.32) | 0.40 | 0.61 (0.33-1.12) | 0.11 |
| 60-64 | 0.92 (0.71-1.19) | 0.51 | 0.68 (0.42-1.10) | 0.11 | 0.61 (0.34-1.10) | 0.10 |
| 65-68 | 0.98 (0.76-1.26) | 0.85 | 1.21 (0.81-1.80) | 0.35 | 0.73 (0.42-1.28) | 0.27 |
| 69-71 | 1.00 (0.78-1.26) | 0.99 | 1.08 (0.72-1.60) | 0.72 | 1.07 (0.67-1.70) | 0.78 |
| 72-74 | 0.65 (0.48-0.87) | <0.01 | 0.60 (0.35-1.02) | 0.06 | 0.73 (0.42-1.28) | 0.27 |
| 75-79 | 0.87 (0.70-1.09) | 0.23 | 1.08 (0.78-1.50) | 0.64 | 1.09 (0.75-1.60) | 0.65 |
| 80-82 | 0.97 (0.76-1.23) | 0.78 | 0.92 (0.63-1.36) | 0.68 | 1.03 (0.67-1.57) | 0.91 |
| 83-87 | 1.37 (1.09-1.72) | <0.01 | 1.26 (0.88-1.82) | 0.21 | 0.97 (0.61-1.54) | 0.89 |
| 88-96 | 1.08 (0.88-1.31) | 0.48 | 1.01 (0.74-1.40) | 0.94 | 1.52 (1.10-2.09) | 0.01 |
| >96 | 1.52 (1.24-1.85) | <0.01 | 1.83 (1.38-2.41) | <0.01 | 2.90 (2.19-3.84) | <0.01 |
|  |  |  |  |  |  |  |

**Supplementary table 9. Multivariable fractional polynomial logistic regression analysis.**

**Sensitivity analysis for heart rate against myocardial injury with 30 days after surgery, corrected for confounding variables. Preoperative heart rate was considered as a continuous variable. Age and eGFR (ml/min) were considered as categorical variables. The remaining covariates were considered as binary categorical variables. The following covariates underwent polynomial transformation: heart rate, (heart rate – 76.68); age, (age - 1.76); preoperative eGFR, ([eGFR^-0.5] – 0.52).**

|  | **Myocardial Injury** | | |
| --- | --- | --- | --- |
| **Covariates** | **odds ratio** | **p-value** | |
| Age (45-64, 65-75, >75) | 1.47 (1.35-1.61) | | <0.01 |
| Male sex | 1.38 (1.20-1.59) | | <0.01 |
| History of atrial fibrillation | 1.53 (1.17-1.99) | | <0.01 |
| History of Diabetes | 1.36 (1.17-1.58) | | <0.01 |
| History of hypertension | 1.30 (1.11-1.52) | | <0.01 |
| History of congestive cardiac failure | 1.60 (1.27-2.00) | | <0.01 |
| History of coronary artery disease | 1.49 (1.26-1.77) | | <0.01 |
| History of peripheral vascular disease | 2.16 (1.76-2.63) | | <0.01 |
| History of stroke or transient ischaemic attack | 1.48 (1.21-1.79) | | <0.01 |
| eGFR (<30, 30-45, 45-60, >60) | 107.30 (71.10-161.92) | | <0.01 |
| History of chronic obstructive pulmonary disease | 1.18 (0.96-1.43) | | 0.12 |
| Neurosurgery | 1.15 (0.88-1.52) | | 0.31 |
| Urgent or emergency surgery | 1.82 (1.54-2.15) | | <0.01 |
| Major Surgery | 1.62 (1.39-1.89) | | <0.01 |
| Heart rate | 1.01 (1.01-1.02) | | <0.01 |
|  |  |  | |

**Supplementary figure 1. Incidence of complications by preoperative heart rate decile.**
